# Suggested Isolation of Wounded Canadians

**Published:** 1916-10-14

**Authors:** 


### SUGGESTED ISOLATION OF WOUNDED CANADIANS.

War-time brings many rumours, and the experienced ear learns to cultivate incredulity. This, we must confess, was our own attitude towards a report put in circulation some few days ago to the effect that the Canadian military authorities intended to collect all wounded soldiers from the Dominion into their own hospitals. The rumour was not merely in the air. On the contrary, it appeared in a letter to the *Times*, signed by a writer of presumed responsibility. Yet, in spite of this imposing show and in spite of the absence of any official contradiction, our incredulity remains. Many experiences have taught us how pleasing to the official mind is a hard, regular, and drilled uniformity, and we cannot therefore deny the possibility that such a proposal as the one above mentioned may perchance have found birth in the brain of a pedant. But that it can have been regarded with favour by the Canadian authorities nothing but actual demonstration will convince us. From abundant evidence we know that both soldiers and civilians at present in this country are strongly opposed to any such course, and protests in this sense are being energetically made in the right quarter. With these we desire to associate ourselves, and we hope without delay to hear either that the report is unfounded or that the suggestion has been abandoned.

The question is not without its national and Imperial side. Hitherto wounded men who have fought for the common cause, from whatever part of the Empire they may have come, have enjoyed in common the hospital service to which it was most convenient to send them. Out of a common suffering and mutual helpfulness have grown opportunities for companionship and comradeship. Britons from home and Britons from overseas have come to know one another in an intimacy which is all the more close from the circumstances which have given it opportunity. The result is an Imperial asset having not only a sentimental interest but also a high practical value. Never before has there been such a sense of oneness between the various communities of the British Empire. Some part of this is certainly due to the recognition in hospitals that we are members one of another. To set up anything like separation, isolation, and detachment is at this moment wholly repugnant to all sympathetic and patriotic citizens, and we await with confidence the announcement that the Canadian authorities have no intention to take any step in this direction.

It is, of course, quite possible that there may be necessities of administration which demand that in the last resort the Canadian soldiers should come into the care of the Dominion authorities. As a practical business arrangement this is probable on the face of things. But while allowing this, it is surely possible to hope that until such need arises all our fighting men who need hospital care may find it in circumstances in which all receive one and the same welcome, and where personal association may lead to a more vivid appreciation of the opportunities and obligations of the British Empire. The time for isolation and detachment is over, and all influences which encourage union and a sense of oneness stand with a strong patriotic defence. The popular instinct is sometimes more to be trusted than official formulæ, and the present issue seems to come under this proposition.

